# Audio-Tactile Integration and the Influence of Musical Training

**DOI:** 10.1371/journal.pone.0085743

**Published:** 2014-01-21

**Authors:** Anja Kuchenbuch, Evangelos Paraskevopoulos, Sibylle C. Herholz, Christo Pantev

**Affiliations:** 1 Institute for Biomagnetism and Biosignalanalysis, University of Münster, Münster, Germany; 2 Montreal Neurological Institute, McGill University, and International Laboratory for Brain, Music, and Sound Research (BRAMS), Montreal, Canada; 3 German Center for Neurodegenerative Diseases (DZNE), Bonn, Germany; Baycrest Hospital, Canada

## Abstract

Perception of our environment is a multisensory experience; information from different sensory systems like the auditory, visual and tactile is constantly integrated. Complex tasks that require high temporal and spatial precision of multisensory integration put strong demands on the underlying networks but it is largely unknown how task experience shapes multisensory processing. Long-term musical training is an excellent model for brain plasticity because it shapes the human brain at functional and structural levels, affecting a network of brain areas. In the present study we used magnetoencephalography (MEG) to investigate how audio-tactile perception is integrated in the human brain and if musicians show enhancement of the corresponding activation compared to non-musicians. Using a paradigm that allowed the investigation of combined and separate auditory and tactile processing, we found a multisensory incongruency response, generated in frontal, cingulate and cerebellar regions, an auditory mismatch response generated mainly in the auditory cortex and a tactile mismatch response generated in frontal and cerebellar regions. The influence of musical training was seen in the audio-tactile as well as in the auditory condition, indicating enhanced higher-order processing in musicians, while the sources of the tactile MMN were not influenced by long-term musical training. Consistent with the predictive coding model, more basic, bottom-up sensory processing was relatively stable and less affected by expertise, whereas areas for top-down models of multisensory expectancies were modulated by training.

## Introduction

Perception of our environment is a multisensory experience as information from different sensory systems like auditory, visual and tactile is constantly integrated. For instance, if we see and hear somebody talk, we process the combined information of mouth/lip movement and speech. A famous example of inconcruency between the auditory and the visual information is the McGurk effect in audiovisual speech perception [1]. It is crucial to unravel the neuronal underpinnings of multisensory processing in order to understand perception as it happens in our natural environment. As sensory processing is modulated by expertise, long-term musical training is an excellent model for brain plasticity driven by the multisensory experience of learning to play a musical instrument. Musical training shapes the human brain on functional and structural levels, affecting a network of brain areas [2]–[4].

The Mismatch negativity (MMN) is an event-related component that reflects the detection of novel sound events that differ from an expected input. Typically, it is elicited in auditory cortex by expectancy violations within simple features (pitch, loudness, timbre) as well as by violations of more complex rules (tone patterns, abstract rules) and has also been shown in paradigms combining various deviant features, the so called multi-feature paradigms [5], [6]. Simple feature MMNs reflecting basic auditory processing are typically not influenced by musical training, while MMN in more complex paradigms reflecting higher-order processing is influenced by musical training [7]–[11]. MMN was originally a phenomenon attributed to the auditory system, but there is evidence from more recent studies demonstrating an MMN response also in other modalities. An MMN-like deflection was found also in the visual system [12] and there are a few studies that report an MMN response in the tactile system [13]–[17].

Musical performance as playing a musical instrument is a multisensory experience involving visual, auditory and tactile percepts. As training shapes the brain, musical training also affects multisensory integration. For example, musical training influences the temporal binding of the senses during perception of audio-visual input in music but not in speech [18]. Also auditory and motor function are closely coupled in music: When musicians listen to music (played by their instrument) the brain areas related to the actual motor task of playing are co-activated [19]–[23]. The focus in these studies was on the motor rather than the tactile aspect of sensorimotor processing, and effects of training on auditory-tactile processing without an overt motor component have not yet been reported.

A recent study from our laboratory used a music-reading paradigm with short melodies, based on a modification of the multi-feature MMN paradigm in order to reveal a cortical response to abstract audio-visual incongruencies [24]. This multisensory response, mainly located in frontal regions, was generated in response to the violation of an abstract rule that binds the auditory and visual stimuli and the response was different from the unisensory auditory and visual MMNs that were also tested in the study. Moreover, it was shown to be modulated by long-term musical training. There are very few studies that investigated combined auditory and tactile MMN. Butler et al. (2012) used EEG to identify multisensory effects from audio-tactile MMNs that were significantly different from the sum of the unisensory auditory and tactile MMNs [25]. Taken together these recent studies using multisensory stimulation suggest that the coupling of sensory information occurs early in the cortical processing, and that musical training has an influence on multisensory integration. Furthermore, multisensory integration reflected in those cortical networks might differ from the unisensory processing.

The goal of the present study was to investigate tactile and auditory MMN during multisensory stimulation and to determine the integration of abstract information from the two senses in an incongruency response that occurs when auditory and tactile stimulation do not match an abstract rule. Moreover, we investigated how long-term multisensory training shapes these neural processes by testing the influence of long-term musical training on audio-tactile processing. We hypothesized that an audio-tactile response is generated in response to the audio-tactile incongruencies, and that this response is different from the unisensory responses. Moreover an influence of long-term musical training on this multisensory response was expected, indicating that training-induced plasticity specifically enhances higher-order processing in musically trained individuals.

## Materials and methods

### Ethics Statement

The study protocol was approved by the ethics committee of the Medical Faculty of the University of Münster and the study was conducted according to the Declaration of Helsinki. No children participated in the experiment; all subjects were at minimum 18 years old and provided written consent prior to their participation in the study.

### Subjects

30 subjects participated in the experiment (15 musicians, mean age: 21.6; SD: 2.44; 4 males; 15 non-musicians, mean age: 29.3; *SD*: 11.93; 7 males). Musicians were students at the Music Conservatory in Münster or professionals or had received extensive musical training since childhood (minimum ten years) and were still actively playing their instrument at the time of study (average practice time of 9 hours per week). None of them had absolute pitch according to self-report and all of them played a string instrument as their principal instrument. Non-musicians were classified by not having received any musical training apart from basic compulsory music classes in school. All subjects were right-handed as assessed by the Edinburgh Handedness Inventory [26] and had normal hearing as assessed by clinical audiometry.

### Stimuli

**Figure 1:**
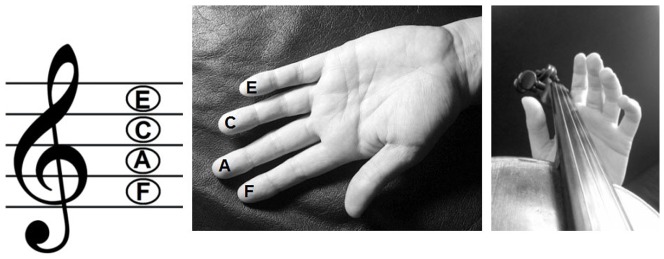
Outline of the multisensory matching rule that defined the audio-tactile correspondence of fingers to pitches.

A paradigm mimicking the tactile and auditory part of musical-instrument playing was set up, combing tones and tactile stimulation with a tone-to-finger relationship as depicted in figure 1. The short melodies used in the present experiment are the same melodies as were used in the study on audio-visual integration by Paraskevopoulos et al. [24], but here they were combined with tactile rather than visual stimuli.

Four different categories of audio-tactile trials (audio-tactile congruent = standard, audio-tactile incongruent, auditory deviant and tactile deviant) were created for setting up the three experimental conditions (audiotactile, auditory and tactile) and the control condition (standard), each consisting of a 5-tone melody and a synchronously presented tactile stimulation to the fingertip (distal phalanges) or the intermediate phalanges (tactile deviant only) of one finger per tone of the left hand (index finger, middle finger, ring finger or little finger, respectively). The five-tone melodies were constructed by a combination of four different sinusoidal tones F5 (698.46 Hz), A5 (880.46 Hz), C6 (1046.50 Hz) and E6 (1318.51 Hz) with duration of 400 ms and 10 ms rise and decay time (44100 Hz stereo, 16 bit). Eight different melodies were composed, each starting with C6. The stimulus onset asynchrony was set to 500 ms and the total duration of each melody was 4 s. Each of the four possible pitches of the melodies corresponded to the tactile stimulation of one of the four fingers of the left hand, starting with the lowest tone (F) corresponding to stimulation of the index finger, the second lowest tone (A) to the middle finger, the second highest tone (C) to the ring finger and the highest tone (E) to the little finger (see figure 1), thus creating a multisensory matching rule of audio-tactile correspondence of fingers to pitches. The ascending order of pitches was modelled after a string instrument analogy.

**Figure 2:**
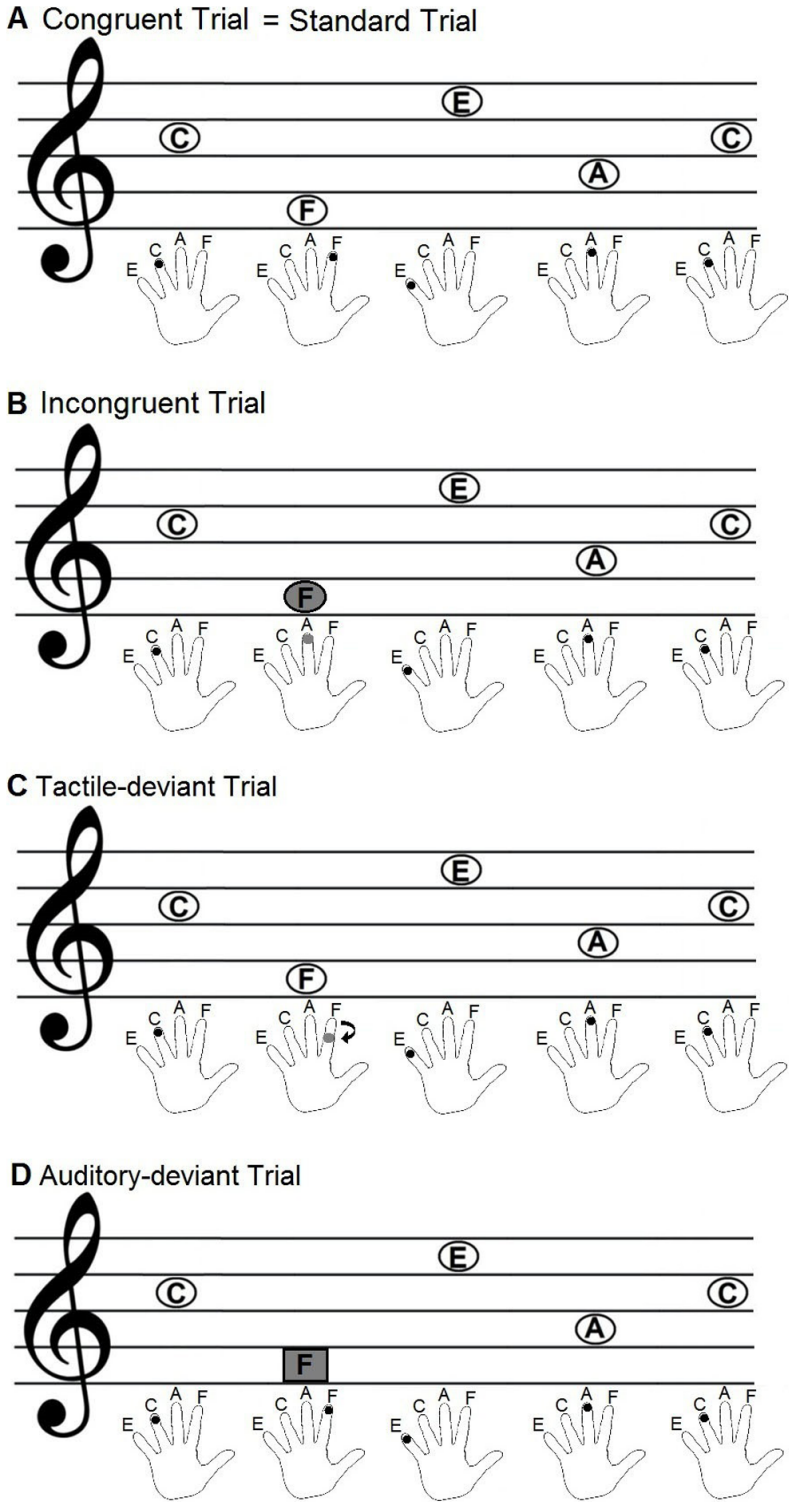
Outline of the four different conditions (A congruent = standard, B incongruent, C tactile deviant and D auditory deviant). The upper part of each image represents the melody played and the lower part shows the exact location of the simultaneous tactile stimulation of one finger of the left hand per tone. Tones in ovals represent sinusoidal timbres and the tone in a rectangle represents a sawtooth timbre. A: In a congruent trial the match of tone and stimulated finger is always correct. B: In an incongruent trial the match of one tone and finger pair is not correct (with regard to the multisensory matching rule). C: In a tactile-deviant trial one time the location of the finger stimulation is shifted from the fingertip to the second phalanx. D: In an auditory-deviant trial one of the tones is presented in sawtooth timbre instead of sinusoidal timbre.

The tactile stimulation was delivered by a pneumatic stimulator via plastic tubes to the fingers of the subjects that created a sensation of touch by a small membrane pressing against the fingertip (or intermediate phalanges) of the subjects. The four different categories of audio-tactile stimuli according to the four different experimental conditions were the following: 1) A congruent audio-tactile trial consisted of congruent matches of audio-tactile stimulation on all 5 tones. This was considered as standard (see figure 2 A). 2) An incongruent audio-tactile trial consisted of 4 congruent and one incongruent finger-pitch pairings. Incongruent means that the tone presented and the finger stimulated were not matching according to the predefined multisensory rule of audio-tactile correspondence of finger to pitch height (see figure 2 B). The violation of this rule could only be identified by the multisensory experience and not by unisensory experience alone. 3) In a tactile deviant trial, the tactile stimulation of the finger in one of the last 4 tones was delivered to the intermediate phalanges instead of the fingertip, but on the correct finger according to the audio-tactile matching rule (tactile deviant, see figure 2 C).) In an auditory deviant trial one of the 4 last tones retained the correct pitch (according to the rule) but had a different timbre created by a saw-tooth waveform filtered with a low-pass filter at 5000 Hz instead of a sinusoidal waveform (auditory deviant, see figure 2 D). In both the auditory and the tactile deviant the multisensory rule was not violated, but the expectancy violation (change of location on finger or change of timbre) occurred within the respective modality. The multisensory, auditory and deviant mismatches occurred equally distributed at one of the last 4 tones of the 5-tone melody.

### Procedure

Stimulus sequences from all 4 categories (total of 32 trials) were presented in random order in each run. Within 2.5 s after each trial the subjects had to indicate via button press with their right hand if the trial was congruent (no button press), incongruent (button press 1), if there was a different tone (auditory deviant, button press 2) or if the tactile stimulation was at the intermediate phalanges instead at the fingertip (tactile deviant, button press 3). During the inter-trial interval, an image was presented to the subjects reminding them which button represented which answer. Instructions about the task and example trials were presented to the participants before the beginning of the MEG recordings. Four runs (length of 14.5 min each) were performed. The total number of stimulus sequences for each category was 128.

### MEG recordings

Magnetic fields were recorded with a 275 channel whole-head system (OMEGA, CTF Systems Inc, Port Coquitlam, Canada) in an acoustically quiet and magnetically shielded room. MEG data were acquired continuously during each run with a sampling rate of 600 Hz. Participants were seated upright, and their head position was comfortably stabilized with cotton pads inside the dewar. The subjects listened to the four runs with short breaks in between, during which they could relax. Auditory stimuli were delivered via air conduction in plastic tubes of 80 cm length at 60 dB SL above the individual hearing threshold, which was determined with an accuracy of 5 dB for each ear at the beginning of each MEG session using the C6 stimulus tone. The tactile stimulation was delivered to the subjects synchronously with the auditory stimulation via a pneumatic stimulator at moderate stimulation intensity. The subject's alertness, well-being and compliance were verified by video monitoring. The subjects were instructed to minimize swallowing and blinking.

### Data analysis

**Figure 3:**
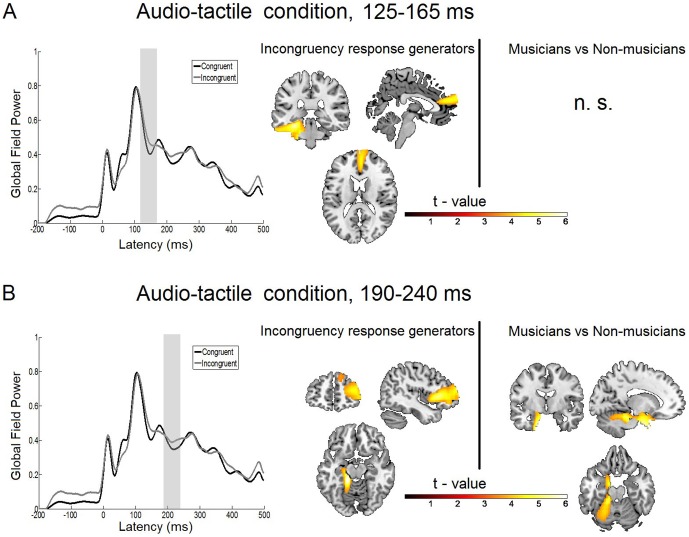
Statistical parametric maps and grand averaged global field power of the audio-tactile incongruency response. A: Right: Statistical parametric maps of the audio-tactile incongruency response and the musicians versus non-musicians comparison as revealed by the flexible factorial model for the time window of 125 to 165 ms. Threshold: AlphaSim corrected at p<0.001 by taking into account peak voxel significance (threshold p<0.001 uncorrected) and cluster size (threshold size,>259 voxels). Left: Grand average global field power for standard (black line) and deviant (grey line) response. The gray bar indicates the time interval where the analysis was performed. B: Right: Statistical parametric maps of the audio-tactile incongruency response and the musicians versus non-musicians comparison as revealed by the flexible factorial model for the time window of 190 to 240 ms. Threshold: AlphaSim corrected at p<0.001 by taking into account peak voxel significance (threshold p<0.001 uncorrected) and cluster size (threshold size,>161 voxels). Left: Grand average global field power for standard (black line) and deviant (gray line) response. The gray bar indicates the time interval where the analysis was performed.

**Figure 4:**
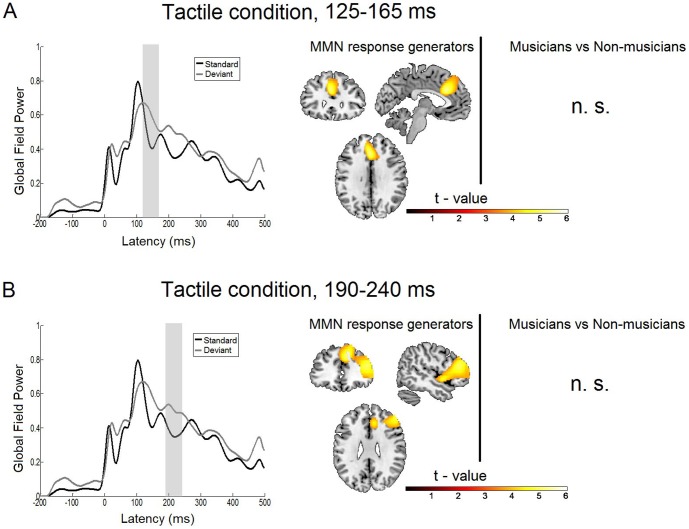
Statistical parametric maps and grand averaged global field power of the tactile MMN response. A: Right: Statistical parametric maps of the tactile MMN response and the musicians versus non-musicians comparison as revealed by the flexible factorial model for the time window of 125 to 165 ms. Threshold: AlphaSim corrected at p<0.001 by taking into account peak voxel significance (threshold p<0.001 uncorrected) and cluster size (threshold size,>198 voxels). Left: Grand average global field power for standard (black line) and deviant (gray line) response. The gray bar indicates the time interval where the analysis was performed. B: Right: Statistical parametric maps of the tactile MMN response and the musicians versus non-musicians comparison as revealed by the flexible factorial model for the time window of 190 to 240 ms. Threshold: AlphaSim corrected at p<0.001 by taking into account peak voxel significance (threshold p<0.001 uncorrected) and cluster size (threshold size,>73 voxels). Left: Grand average global field power for standard (black line) and deviant (gray line) response. The gray bar indicates the time interval where the analysis was performed.

**Figure 5:**
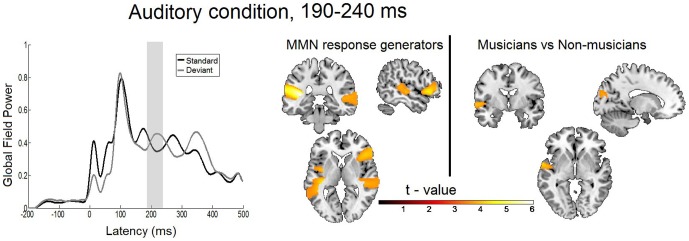
Statistical parametric maps and grand averaged global field power of the auditory MMN response. Right: Statistical parametric maps of the auditory MMN response and the musicians versus non-musicians comparison as revealed by the flexible factorial model for the time window of 190 to 240 ms. Threshold: AlphaSim corrected at p<0.001 by taking into account peak voxel significance (threshold p<0.001 uncorrected) and cluster size (threshold size,>197 voxels). Left: Grand average global field power for standard (black line) and deviant (gray line) response. The gray bar indicates the time interval where the analysis was performed.

The Brain Electrical Source Analysis software (BESA Research, version 5.3.7; Megis Software) was used to pre-process the MEG data. The recorded data were separated into epochs of 700 ms, including a prestimulus interval of 200 ms and a poststimulus interval of 500 ms. Epochs containing MEG signals larger than 2.5 pT were considered artifact-contaminated and were excluded from averaging. The data was filtered offline with a high-pass filter of 1 Hz and a low-pass filter of 30 Hz. Epochs were baseline-corrected using the interval from −100 to 0 ms. Averages of all four runs were computed separately for the congruent and the incongruent stimuli of the audio-tactile modality and for the deviants of the auditory and tactile modalities. All stimuli of the congruent trials that were not timbre or tactile deviant were used as standards in all comparisons. The incongruent, auditory and tactile deviants were the corresponding stimulus events in incongruent and deviant trials, resulting in a deviant-to-standard ratio of 1∶4. Current density reconstructions (CDR) were calculated on the neural responses of each subject for each stimulus category (congruent audio-tactile, incongruent audio-tactile, auditory deviant, tactile deviant) using the low-resolution brain electromagnetic tomography (LORETA) method [27], as implemented in BESA software. With the LORETA method a current distribution throughout the full-brain volume is calculated. This method has the advantage of not needing an a priori definition of the number of activated sources. The averaged global field power (GFP) was computed separately for each modality (see figures 3–5). Based on the grand average results of the GFP computation two time windows were chosen for the analysis, which contained a stronger activation in the deviant or incongruent than in the standard conditions and which were within the typical latency window of MMN (ranging from 110–250 ms) [28]. An early time window of 40 ms (125–165 ms), which contained stronger activation in the deviant of the audio-tactile and tactile but not the auditory modality, was chosen for the audio-tactile and the tactile condition. A later time window of 50 ms (190–240 ms), which contained stronger activation in the deviant in all three modalities, was chosen for all three modalities. Each individual's mean CDR image within the selected time window was calculated and projected onto a standard MRI template based on the Montreal Neurological Institute (MNI) template. The images were smoothed and their intensities normalized by convolving an isotropic Gaussian kernel with 7 mm full width half-maximum through BESA's smoothing utility. Statistical Parametric Mapping 8 software (SPM8, http://www.fil.ion.ucl.ac.uk/spm) was used for the statistical analysis of the CDRs. Using second level analysis of SPM, a separate flexible factorial model was computed for each modality (audio-tactile, auditory, and tactile) to explore the main effect of condition (deviant/incongruent to standard comparison) and the group×condition interaction. The flexible factorial model is SPM's equivalent to a 2×2 mixed-model ANOVA with between-subject factor group and within-subject factor condition. The factors included in the analysis were subject, group (musicians and non-musicians), and condition (standard and deviant/incongruent). A mask was used to constrain the results to gray matter, including the cerebellum, thereby keeping the search volume small and in physiologically reasonable areas. A permutation method for peak-cluster level error correction (AlphaSim) at 5% level was applied for this analysis, as implemented in REST software [29] so that the significance of the peak voxel (threshold, p<0.001 uncorrected) along with the appropriate cluster size for each analysis (audio-tactile early time window, audio-tactile late time window, tactile early window, tactile late window and auditory late window) was taken into account, thereby controlling for multiple comparisons. All anatomical labeling was based on the Jülich atlas [30] and the cerebellar atlas by Diederichsen et al [31].

## Results

### Behavioral Results

The behavioral results were evaluated in percent correct for the identification of an incongruent trial, an auditory deviant trial and a tactile deviant trial. Due to technical and procedural reasons the behavioral responses differed between conditions (audio-tactile congruent required no button press while every other condition required a button press of one out of 3 buttons). Hence the discriminability index d′ and the difference of the averaged answers from chance level could not be calculated. The behavioral results were entered in percent correct in a repeated-measures 2×3 ANOVA with between-subject factor group (musicians and non-musicians) and within-subject factor condition (incongruent, tactile deviant and auditory deviant). Results show significant main effects for condition [F (2,56) = 20.882, p = .000] and group [F (1,28) = 5.482, p = .027] as well as a significant interaction of group×condition [F (2,56) = 3.713, p = .031].

**Figure 6:**
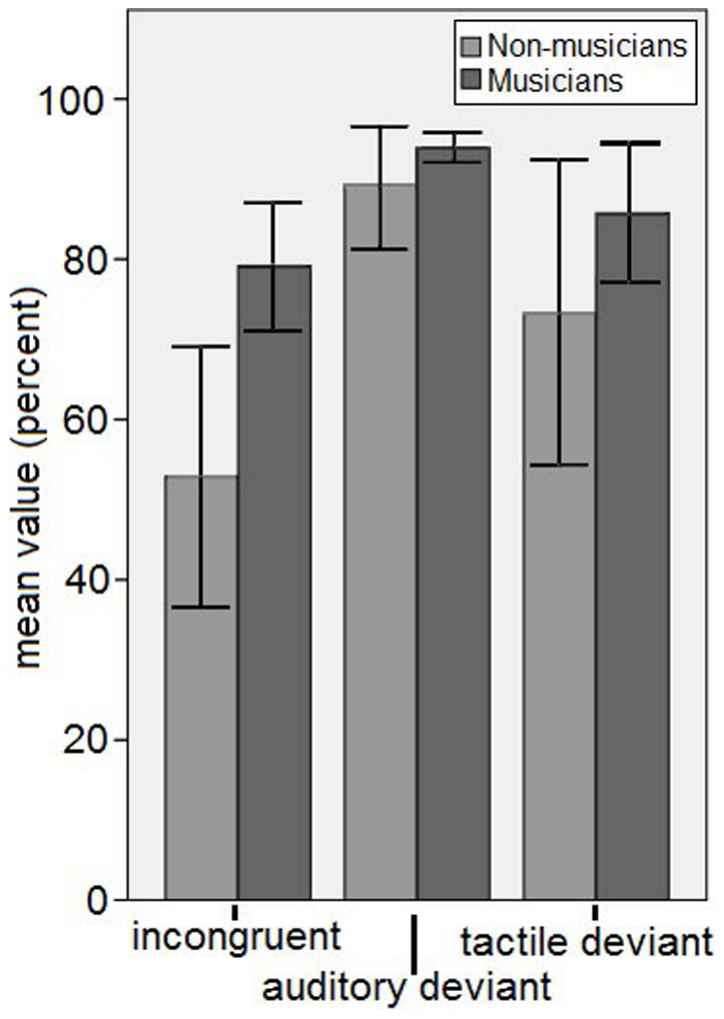
Behavioral results of the correct answers in percent, plotted separately by group (musicians in dark grey and non-musicians in light grey) and condition (incongruent, congruent, auditory deviant and tactile deviant, x-axis). Error bars represent the 95% confidence interval.

Post-hoc t-tests showed that the percentage of correct answers was significantly higher in the auditory condition than in all other conditions and that the percentage of correct answers was significantly higher in the tactile condition than in the incongruent condition. According to these results the incongruent condition was the most difficult to identify. Musicians (79±14% SD) showed a significantly higher percentage of correct answers than the non-musicians (53±29% SD) in the incongruent condition [t(28) = −3.118, p = .004, independent samples t-test]. The percentage of correct answers in the musicians was also higher than in the nonmusicians for the other two conditions, but not significantly so. The behavioral results are shown in percent of correct answers for every condition separated by group (figure 6).

### MEG data: Audio-tactile condition

#### Incongruency response generators, time window 125–165 ms

**Figure 7:**
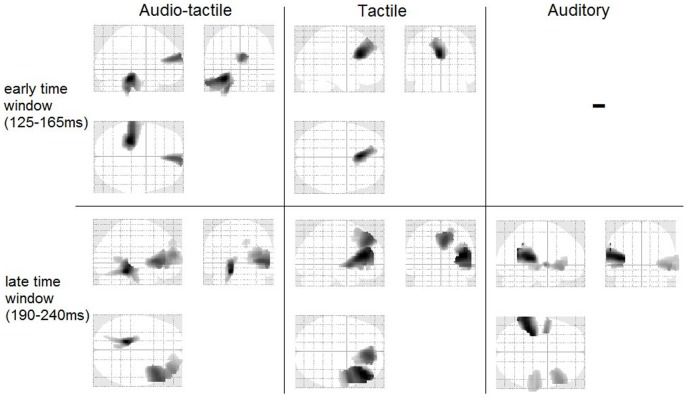
Glass brain view of activations for the main effects of all conditions (audio-tactile, tactile, and auditory) and both time windows (125–165 ms and 190–240 ms).

Statistical analysis of the main effect of the audio-tactile incongruency response [condition (audio-tactile incongruent>audio-tactile congruent)] for the earlier time window of 125 to 165 ms revealed two main generators located in left temporal and frontal regions. Specifically, the first effect was a broad region reaching from the left parahippocampal gyrus (z = 18; t(28) = 5.55; cluster size = 3138 voxels; p<0.001 AlphaSim corrected) over the left inferior temporal gyrus to the left cerebellum. The second effect was generated in a medial frontal region with its peak in the right medial frontal gyrus (t(28) = 4.72; cluster size = 942 voxels; p<0.001 AlphaSim corrected). These results are listed in table 1 and the statistical map is presented in figure 3A. The overview of the activations of all conditions and both time points is also presented in a transparent brain in figure 7.

**Table 1:** Generators of the incongruency response of the audio-tactile modality and the MMN responses of the tactile modality in the time window 125–165 ms.

Modality	Location of activation	MNI Coordinates			Peak voxel *t* value	Cluster size
		X	Y	Z		
Audio-tactile	Left Parahippocampal Gyrus	−30	−30	−18	5.55	3138
	Left Inferior Temporal Gyrus	−54	−26	−24	4.78	
	Left Inferior Temporal Gyrus	−62	−26	−26	4.70	
	Right Medial Frontal Gyrus	4	54	18	4.72	942
	Right Medial Frontal Gyrus	4	62	22	4.70	
Tactile	Left Anterior Cingulate	−2	26	28	5.44	2064
	Left Medial Frontal Gyrus	−10	44	42	4.49	
	Left Superior Frontal Gyrus	−20	48	46	3.85	

#### Incongruency response generators, time window 190–240 ms

In the statistical analysis of the main effect of the audio-tactile incongruency response (condition) for the later time window (190 to 240 ms) we found left temporal activation and right-lateralized large frontal activation. Specifically, the broadest activation was found to be in the right inferior frontal gyrus (t(28) = 5.37; cluster size = 4623 voxels; p<0.001 AlphaSim corrected) extending to the right medial frontal gyrus and a smaller area in the right superior frontal gyrus t(28) = 3.62; cluster size = 189 voxels; p<0.001 AlphaSim corrected), which was not connected to the first one. The strongest activation was found deeper in temporal areas, close to the left cerebellum (t(28) = 6.27; cluster size = 1495 voxels; p<0.001 AlphaSim corrected) extending to the left fusiform gyrus and the left parahippocampal gyrus. These results are displayed in table 2 and figures 3B and 7.

**Table 2:** Generators of the incongruency response of the audio-tactile modality and the MMN responses of the auditory and tactile modalities in the time window 190–240 ms.

Modality	Location of activation	MNI Coordinates			Peak voxel *t* value	Cluster size
		X	Y	Z		
Audio-tactile	Left Fusiform Gyrus	−16	−36	−16	6.27	1495
	Left Parahippocampal Gyrus	−14	−32	−8	5.75	
	Left Cerebellum V	−8	−46	−8	3.95	
	Right Inferior Frontal Gyrus	46	24	4	5.37	4623
	Right Medial Frontal Gyrus	40	54	14	4.61	
	Right Inferior Frontal Gyrus	46	42	2	4.54	
	Right Superior Frontal Gyrus	16	58	38	3.62	189
	Right Superior Frontal Gyrus	20	52	42	3.46	
Auditory	Left Transverse Temporal Gyrus	−32	−30	10	6.07	2749
	Left Transverse Temporal Gyrus	−38	−36	10	6.03	
	Left Superior Temporal Gyrus	−52	−40	16	5.84	
	Left Claustrum	−36	−2	0	4.64	363
	Left Superior Temporal Gyrus	−56	2	−2	4.26	
	Right Inferior Frontal Gyrus	54	24	−4	4.38	1165
	Right Medial Temporal Gyrus	66	−18	−8	4.03	1520
	Right Superior Temporal Gyrus	44	−30	4	3.80	
Tactile	Right Inferior Frontal Gyrus	46	36	16	5.61	4482
	Right Inferior Frontal Gyrus	40	30	14	5.57	
	Right Medial Frontal Gyrus	46	46	24	5.34	
	Right Medial Frontal Gyrus	8	44	46	4.96	2473
	Right Superior Frontal Gyrus	20	40	54	4.56	
	Right Superior Frontal Gyrus	22	48	46	4.47	

#### Musicians versus non-musicians comparison

The interaction effect (incongruency×musical training) was not statistically significant in the earlier time window. In the late time window we found an interaction effect with musicians showing a greater incongruency response than non-musicians in a large cluster stretching along the left uncus (t(28) = 5.42; cluster size = 3062 voxels; p<0.001 AlphaSim corrected), left premotor gyrus and left cerebellum (see table 3 and figure 3B).

**Table 3:** Location of activity in musicians vs. non-musicians comparison in the time window 190–240 ms.

Modality	Location of activation	MNI Coordinates			Peak voxel *t* value	Cluster size
		X	Y	Z		
Audio-tactile	Left Uncus	−18	−6	−36	5.42	3062
	Left Premotor Cortex^1^	−16	2	42	5.36	
	Left Cerebellum V	−16	−36	−20	5.03	
Auditory	Left Superior Temporal Gyrus	−60	−2	−2	4.08	264
	Left Cuneus	−16	−86	22	3.71	204
	Left Medial Occipital Gyrus	−22	−96	16	3.41	
Tactile	n. s.					

1 Closest labeled region (2 mm distance) according to the Jülich Atlas.

### MEG data: Tactile condition

#### MMN response generators, time window 125–165 ms

The statistical analysis of the main effect (condition) of the tactile deviant response for the earlier time window of 125 to 165 ms revealed a frontal activation extending over the left anterior cingulate gyrus (t(28) = 5.42; cluster size = 2064 voxels; p<0.001 AlphaSim corrected), the left medial frontal gyrus and the left inferior frontal gyrus (see table 1 & figures 4A and 7).

#### MMN response generators, time window 190–240 ms

In the later time window the activation included two regions: the right inferior/medial frontal gyrus (IFG, t(28) = 5.61; cluster size = 4482 voxels; p<0.001 AlphaSim corrected) as well as the right medial/superior frontal gyrus (t(28) = 4.96; cluster size = 2473 voxels; p<0.001 AlphaSim corrected). These results are shown in table 2 and figures 4B and 7.

#### Musicians versus non-musicians comparison

No significant interaction effects were found in the tactile modality between musicians and non-musicians.

### MEG data: Auditory condition

#### MMN response generators, time window 190–240 ms

The statistical analysis of the main effect of the auditory deviant response for this time window revealed a network of bilateral activation in temporal regions along with right frontal activation. Specifically, activations were found in the left transverse/superior temporal gyrus (TTG; t(28) = 6.07; cluster size = 2749 voxels; p<0.001 AlphaSim corrected) and left claustrum (t(28) = 4.94; cluster size = 363 voxels; p<0.001 AlphaSim corrected), in the right inferior frontal gyrus (t(28) = 4.38; cluster size = 1165 voxels; p<0.001 AlphaSim corrected) and right medial/superior temporal gyri (t(28) = 4.03; cluster size = 1520 voxels; p<0.001 AlphaSim corrected). These results are displayed in table 2 and figures 5 and 7.

#### Musicians versus non-musicians comparison, time window 190–240 ms

Significant interaction effects were found for musicians having greater MMN activation than non-musicians in a small cluster in the left superior temporal gyrus (t(28) = 4.08; cluster size = 264 voxels; p<0.001 AlphaSim corrected) and in a region of the left cuneus (t(28) = 3.71; cluster size = 204 voxels; p<0.001 AlphaSim corrected) extending to the left medial occipital gyrus (c.f. figure 5 and table 3).

## Discussion

In the present study patterns of auditory and tactile stimuli were presented to a group of musicians and a group of non-musicians. The subjects had to identify if the auditory and tactile stimuli were congruent or incongruent and if the stimulation pattern included a tactile or an auditory within-modality deviant. The behavioral results show that the audio-tactile incongruencies were hardest to identify for both groups, but that the musicians were better in identifying them than the non-musicians, probably due to their long-term musical training, which is inherently multimodal.

The discussion of the MEG results is divided into two sections. We will first discuss the unisensory MMN responses and activation related to modality-specific processing. Briefly, the tactile mismatch source localization derived from the tactile condition was independent of musical expertise, and in the auditory condition we identified activation that is in line with the known sources of MMN. Secondly, we discuss the findings that can be attributed to the integration of the two modalities, along with group differences attributed to musical expertise. Briefly, the results from the audio-tactile condition include areas that seem to be driven by the tactile stimuli, along with areas that indicate integration of the auditory and tactile information. While the former can be attributed to bottom-up processing and were unaffected by musical expertise, the latter integration areas were the areas that were also influenced by musical expertise.

### Unisensory MMN responses and activation related to modality-specific processing

In the main effect of the auditory MMN condition we see a network of bilateral activation in temporal regions and right inferior frontal activation. The temporal activation indicates an auditory MMN response which is in line with the pertinent literature [32], [33]. The frontal region activation may be attributed to the attention changes for detecting the auditory deviant when switching from multisensory audio-tactile processing to the uni-sensory auditory modality [24] rather than to the frontal source of MMN that is difficult to detect due to blindness of MEG to sources with radial orientation [34].

In the group comparison (interaction effect) musicians showed enhanced activation in small areas in the left superior temporal gyrus and in a region of the left cuneus expanding to the left medial occipital gyrus. Similar enhanced activation of the superior frontal gyrus in musicians with the same auditory stimuli has been found in the study of Paraskevopoulos et al. (2012). In both studies the auditory deviant tone was presented in a rather unpleasant sawtooth timbre (compared to the pure sinusoidal standard tones), which may have induced the activation in the superior frontal gyrus, an area that is activated while listening to unpleasant music [35]. The fact that musicians showed an enhanced activation in superior temporal gyrus may be related to an enhanced sensibility of musicians to unpleasant musical stimuli and timbres [24], [36]. As noted above, the stimulation in the present paradigm is always multimodal and required the attention and expectation-based decision-making of the subjects. This may also explain the strong contribution of frontal areas, which have been shown to be generally involved in attention-shifting due to task-switching [37], [38].

The stronger activation of medial occipital cortex in musicians than nonmusicians was unexpected as this region is mainly related to visual processing, whereas the stimulation in the current paradigm was audio-tactile. However, a strong binding of the auditory, tactile and visual systems might have developed in the highly trained musicians due to their experience in music reading. Therefore, recruitment of some parts of this network might evoke activity also in the rest part of the network that is not directly activated. Comparable cross-modal co-activations have been found across other sensory domains: activation of premotor areas has been found in an audio-visual music-reading-like paradigm [24] and other music reading studies [39], [40].

Previous studies on the tactile MMN often found two event related components, with the first one peaking around 100–200 ms and the later one peaking around 170–270 ms [15]–[17], [41], [42]. Likewise, we find also two components in the tactile condition of the present study. While previous studies did not perform source localization of these components, our results help to attribute the two components to distinct neural generators. The initial component of the tactile mismatch response (early time window 125–165 ms) is located in a medial frontal region touching the anterior cingulate, medial frontal and superior frontal gyri. Albeit the peaks are localized on the left, the whole activation is rather medial, with contributions of both left and right hemispheres (see figure 4A and table 1). In the later component (later time window) the activity extends more to the right (contralateral to the stimulation), forming two broader activations, one in an inferior and medial frontal gyrus and the other one in the superior frontal gyrus (figure 4B and table 2). These activations may be part of a network reflecting the sources of the tactile MMN, which, to our knowledge, have not been localized until now. The task in the present study in the tactile condition was to detect the tactile deviants - a spatial discrimination task with a temporal component involving attention-switching and decision-making. The areas activated by tactile deviants in the present study have been found to be part of a network for more complex tactile processing like tactile object recognition [43]. The anterior cingulate gyrus seems to be especially involved in tactile temporal discrimination tasks compared to a pure detection task [44]. The prefrontal cortex, particularly the left dorsolateral prefrontal cortex, and the dorsal anterior cingulate cortex play a major role in tactile decision making [45].

In the audio-tactile condition (audio-tactile incongruency response) a comparable frontal region is activated: In the early time window we see an activation in a medial frontal region (figure 3A), which is similar to the one described above for the early window in the tactile condition, albeit more superior, stretching to the frontal pole. Likewise in the late time window of the audio-tactile condition we see a shift from the frontal activation in the early time window to a more right lateralized activation, which is a similar pattern as in the tactile condition. The two activations are located in right inferior/medial frontal and right superior frontal regions (figure 3B) similar to the ones described for the tactile condition, although the regions in the tactile condition extend deeper into the inferior frontal gyrus. These patterns are distinct from the sources of the auditory MMN. Therefore it seems plausible that some part of the frontal activity in the audio-tactile condition is more strongly driven by the tactile than by the auditory component of the stimulation. In this frontal region, activated in the tactile and in the audio-tactile condition no differences were found between non-musicians and musicians. Therefore one may assume that the frontal regions belong to the cortical network for basic sensory, bottom-up processing that are not differently activated at varying expert level. Similarly, in the MMN literature for auditory processing it has been shown that the MMN amplitude in basic processing like pitch discrimination is not modulated by long-term musical training [11], [46].

### Integration of the two modalities

Regions that are activated in the audio-tactile but neither in the tactile nor auditory conditions include the more anterior part of the frontal cortex and a network including cerebellum, fusiform gyrus and parahippocampal gyrus. The fact that these activations are distinct from the patterns seen for unisensory discrimination processing indicates their special role in integrated, abstract processing of audio-tactile multisensory input. Multiple association areas within frontal cortex are involved in higher-order or executive functions. The frontal pole of the frontal cortex (especially the anterior part, Brodmann's area 10) is suggested to be involved in cognitive branching, a function described as maintaining a previously running task in a pending state for subsequent retrieval and execution [47]. This frontal pole function of simultaneous engagement in multiple tasks and their integration has also been shown to correlate with abstract reasoning [48]. Moreover, this region has been shown to integrate various information sources in order to guide appropriate actions to a goal [49]. Because of its connections to other brain areas, like higher-order association cortical areas along with auditory and multisensory regions of the superior temporal sulcus, it has been associated with multisensory integration (visual, auditory and somatosensory [50], [51]). In the present study during the audio-tactile condition different sensory information had to be combined in order to form a decision based on an abstract congruency rule. The activation of the frontal pole suggests an active role of it in the integration process and in the combination of the different sensory information, related to the goal of detecting the multisensory rule violations.

Apart from the frontal regions another area is activated in the audio-tactile condition (late time window), which is present neither in the tactile condition nor in the auditory condition. Specifically, this area stretches from the fusiform gyrus over the left parahippocampal gyrus to the left cerebellum (figure 3B). The cerebellum is a region known to be involved in motor control and fine-tuning and calibration of movement parameters such as coordination, precision, and accurate timing during movement execution. A recent study [52] recorded somatosensory MMNs in controls and patients with cerebellar lesions and observed clearly abnormal somatosensory MMNs in patients in the affected hemisphere, while other recorded ERPs, for example during a standard-omitted condition, were normal. These data demonstrate the contribution of the cerebellum in somatosensory input change processing. The cerebellum also plays a major role in implicit learning and procedural memory. Automatic movements such as moving face muscles when speaking or moving the finger when playing a musical instrument are partly stored in the cerebellum [53]. There is growing evidence that sensory-motor networks also contribute to other high-level cognitive functions such as auditory working memory. A recent fMRI study by Schulze et al. (2011) shows the contribution of cerebellum, premotor cortex and other sensori-moror related areas in tonal but not verbal auditory working memory [54]. Functional imaging studies also have shown cerebellar activation in mental imagery: a study with professional and amateur violin players comparing actual playing and imagery of playing music show similar networks including cerebellar activation in both playing and imagery [55], thus indicating a recruitment of stored movement programs also during imagery. Furthermore, in the above-mentioned study the professional violin players revealed more anterior cerebellar activations than the amateurs.

In the present study the subjects were not actually playing an instrument during the experiment. Instead the audio-tactile stimulation was similar to sensory input during instrument playing. The fact that the observed activation in the left fusiform gyrus, parahippocampal gyrus and cerebellum is only present in the multisensory condition thus indicates an important role of these regions in early audio-tactile processing.

The fact that the inconcruency response (audio-tactile processing) involved different brain regions than the unisensory auditory and tactile processing may be a sign of hierarchical organization. On a more conceptual level the results can be interpreted in the context of the predictive coding theory that has also been applied to the MMN [33], [56], [57]. In predictive coding, basic sensory input is constantly compared to predictions from higher-level areas. The violation of an expected event causes a prediction error that results in an adjustment of the higher-order model. In the context of our study the audio-tactile matching rule represents a more complex internal model than the basic expectancies regarding stimulus location and timbre in the unisensory MMNs. Our results correspond to this theory in that the more complex rule violations elicited distinct brain activity in brain regions related to complex, higher-order cognition.

Furthermore, we observed a clear influence of musical training on the networks for audio-tactile integration: the musicians compared to the non-musicians show an increased activation in the left hemisphere including cerebellum, uncus, and premotor cortex, which is consistent with findings of increased activity in these areas during complex multisensory musical cognition [55]. This also corresponds to the previously discussed role of the cerebellum in multisensory (audio-tactile) processing, and the present results suggest that its activity in response to multisensory stimuli is modulated by expertise.

Recently, Vuust et al. (2009) described that musical training affects the neuronal networks involved in rhythm processing relying on a better top-down model (the meter) for the expected stimuli and therefore the predictive coding model [58]. Consistently, our results indicate that expertise has a stronger influence on higher order levels of processing than on bottom up processing, and we extend this conclusion to multisensory integration. Musicians may have a better internal model for the correspondence of information from multiple senses (the representation of playing of a musical instrument) or may more easily adjust such an internal model due to short-term experience in the experiment. These potential advantages through previous experience seem to enhance the top-down processing between levels of different hierarchy in multisensory integration.

## Conclusions

The present study reveals the neural correlates of an integrative audio-tactile incongruency response that are partly overlapping and partly distinct from sources of unisensory auditory and tactile MMN responses. While overlapping activity seems to represent basic bottom-up processing of sensory information, distinct patterns of activation relate to internal models and higher-order multisensory processing. Musicians show an enhanced multisensory incongruency response as well as an enhanced auditory MMN, indicating plasticity effects of musical training on multisensory integration and the processing of complex auditory stimuli, whereas musical training did not affect the tactile MMN. The obtained results suggest that musical training enhances higher-order or top-down processing with a particular emphasis on multisensory integration, whereas more basic processing is relatively less changed. This is consistent with predictive coding theory where bottom-up processing is assumed to be rather stable, whereas higher-order internal models are assumed to change through experience.
